# *MiR-100* rs1834306 A>G Increases Biliary Atresia Risk in Southern Han Chinese Children

**DOI:** 10.1155/2023/4835839

**Published:** 2023-01-04

**Authors:** Jiaming Chang, Jiankun Liang, Chengwei Chai, Fei Liu, Boyuan Tao, Hezhen Wang, Yanlu Tong, Zhe Wang, Huimin Xia

**Affiliations:** Department of Pediatric Surgery, Guangzhou Institute of Pediatrics, Guangzhou Women and Children's Medical Center, Guangzhou Medical University, Guangzhou, 510623 Guangdong, China

## Abstract

**Background:**

Biliary atresia (BA) is a type of severe cholestatic childhood disease that may have a genetic component. *miR-100* plays a key role in regulating cell apoptosis, proliferation, and inflammatory reactions. A single-nucleotide polymorphism in *miR-100* has been proven to modulate susceptibility to various diseases.

**Methods:**

We conducted a case-control retrospective study to explore the correlation between *miR-100* gene polymorphism (rs1834306 A>G) and biliary atresia susceptibility in 484 Chinese patients and 1445 matched control subjects.

**Results:**

Our results showed that rs1834306 A>G was correlated with a significantly increased risk for BA (GG vs. AA: adjusted odds ratio (OR) = 1.44, 95%confidence interval (CI) = 1.02–2.03, *p* = 0.041; and GG vs. AA/AG: adjusted OR = 1.39, 95%CI = 1.02–1.89, *p* = 0.036).

**Conclusions:**

Our results showed that the rs1834306 A>G polymorphism is associated with an increased risk for BA and contributes to BA susceptibility.

## 1. Introduction

Biliary atresia (BA), a type of severe cholestatic disease, occurs in neonatal infants and leads to progressive hepatic fibrosis and hepatitis failure [1, 2]. The incidence of biliary atresia varies among different populations, and it is noteworthy that Asians have a high prevalence of this disease: 1.04/10000 in Japanese and 3.7/10000 in Taiwanese [3]. If left untreated, the prognosis in patients with BA will be extremely poor, with 100% mortality from end stage of liver disease within two years [4]. The Kasai portoenterostomy is the main surgical treatment for BA [5]; nevertheless, transplantation of liver is required for treating end stage of liver disease after operation [6]. Treatment in its earliest stages can effectively prevent progression of liver fibrosis [7], thus early screening and diagnosis are of key importance for efficient treatment [8]. The etiology and pathogeny of BA remain unclear [9].

The influence of antenatal exposure to environment factors such as cytomegalovirus (CMV), intraamniotic infection, and medication therapy on BA pathogenic mechanisms is uncertain [10]. Accumulating evidence demonstrated that genetic element might be of key importance for the pathogenesis of BA [11]. Genome-wide association studies (GWASs) have found that certain genes, such as *ADD3* and *XPNPEP1*, may increase a person's susceptible to BA [12, 13]. Moreover, large amounts of genes with single-nucleotide polymorphisms (SNPs) are worthy to be studied for their association with BA.

*miR-100*, a crucial member of the microRNA- (miR-) 99 family, has been studied in many research. And previous studies have shown its participation in regulating cellular apoptosis and proliferation [14]. Besides that, *miR-100* is also implicated in the modulation of immunocyte response and multiple biological functions, and thereby may play a crucial part in relieving inflammation reaction and maintaining tissular homeostasis [15]. To the best of our knowledge, up to now, no correlation between the *miR-100* gene and BA has been reported. Considering the importance of the *miR-100* gene in cellular metabolic activities and tissue immunological microenvironment, we carried out a GWAS in a group of ethnic homogeneity patients (484 case patients and 1445 control subjects) from the largest Southern Chinese Han nationality. This study was to investigate the association between *miR-100* gene polymorphism and BA susceptibility in Southern Chinese Han children. Our results provide theoretical genetic guidance for further mechanical investigations and may aid in selecting a biomarker for use in the early detection of populations at high risk for biliary atresia.

## 2. Materials and Methods

### 2.1. Study Population

Totals of 484 BA patients and 1445 control subjects were included in this research. These patients were recruited from the Guangzhou Women and Children's Medical Center. All samples were histopathologically confirmed by a pathologist, and research programs was validated by the Institutional Review Board of Guangzhou Women and Children's Medical Center. As per relevant laws and regulations, written medical informed consent document was acquired from each participant's legal guardians. This study was conducted strictly observe all instructions to insure its reliability, precision, and repetitiveness.

### 2.2. SNP Selection and Genotyping

The SNPinfo (http://snpinfo.niehs.nih.gov/snpfunc.htm) and dbSNP database (http://www.ncbi.nlm.nih.gov/SNP) were applied for screening possible function-related polymorphic sites in the *miR-100* gene. TaqMan real-time fluorescence PCR was applied for genotyping the *miR-100* rs1834306 A>G. To help ensure the reliability of our results, all staff members were blinded to sample information. Moreover, 10% of the specimens were selected in random for repeatedly genotyped experiments, and all results were found to be 100% reproducible.

### 2.3. Statistical Analysis

The distribution of sample characteristics between the patient and matched control groups was analyzed by a two-sided chi-squared testing. The consistency between Hardy–Weinberg equilibrium (HWE) and the genotype frequencies in matched control group was identified by a chi-squared goodness-of-fit testing. Multivariate logistic regressive analysis was used in calculating odds ratios (ORs) and 95% confidence intervals (CIs), and also in assessing the interrelation between *miR-100* rs1834306 A>G and the risk for BA. All statistical analyses were conducted using SAS 9.5 software (SAS Institute Cary, NC, the USA). It is considered to be statistically significant when a *p* value <0.05.

## 3. Results

### 3.1. Population Characteristics

As shown in Table 1, this study enrolled 484 BA patients and 1445 control subjects. BA was diagnosed by cholangiography, and the control subjects were children with no hepatobiliary disease.

### 3.2. Associations between *miR-100* Gene Polymorphism and Biliary Atresia Susceptibility

We successfully genotyped 484 biliary atresia patients and 1445 control subjects. The genotype frequencies of the *miR-100* rs1834306 A>G polymorphism in BA patients and control subjects are summarized in Table 1. The frequency distribution of the *miR-100* rs1834306 A>G genotype adhere to the HWE genetic balance in the control group (*p* = 0.126). Most importantly, *miR-100* rs1834306 A>G was found to be significantly associated with an increased risk for BA (GG vs. AA: adjusted OR = 1.44, 95%CI = 1.02–2.03, *p* = 0.041; and GG vs. AA/AG: adjusted OR = 1.39, 95%CI = 1.02–1.89, *p* = 0.036) after adjusting for gender and age.

## 4. Discussion

*MiR-100* is one member of the microRNA- (miR-) 99 family and related with the apoptosis, invasion, proliferation, and tumor cell differentiation [15, 16]. This molecule is evolutionarily conserved, indicating that it plays an indispensable role in regulating various gene expression processes [17, 18]. Pre-miRNA is an important progenitor molecule of miRNA and can cause the abnormal expression of various miRNAs, which could further alters the regulation of a targeted mRNA by miRNA, eventually resulting in a number of diseases [19–21]. The miR-100 rs1834306 A>G polymorphism is located in the pre-miR-100 molecule. Editing function of miR-100 is associated with several biologically relative target genes [22], including *MTOR*, *SMAD2*, *FOXA1*, and *Myc* [23–26]. As a result, it can regulate the intracellular cycle, tissular inflammation, and cellular proliferation [27–29]. According to previous reports, the *miR-100* gene take part in the notch pathway and activates *HES1* [30], indicating that it is an important regulatory factor in developmental disabilities [31] and the cellular growth cycle [32, 33]. It is worth mentioning that the notch pathway was shown to regulate BA pathogenesis in our previous study [34]. In addition, the *miR-100* gene is also involved in the inflammatory response. In other words, *miR-100* gene may provide a chronic inflammatory liver microenvironment for the pathogenesis of BA by influencing the differentiation and activity of various immune cells [23, 35, 36].

A number of studies have shown that the *miR-100* gene takes part in the pathogenesis of many diseases. According to past studies, Jones et al. [37] pointed out that *miR-100* is significantly upregulated in age-related fracture/osteoporosis and shows promise as a biomarker for that disease. Shukla et al. [38] mentioned that its important role in wound repair and interaction with specific genes makes *miR-100* a promising marker. A study by Yang et al. [39] suggested that *miR-100* might be of key in regulating the radiotherapy sensitivity of radioresistant colorectal carcinoma, and could potentially serve as a new clinical target for radiation therapy. *miR-100* rs1834306 was recently reported to be associated with a diminished risk for esophageal squamous epithelial cell cancer in northwestern Chinese Kazakh patients [40]. Furthermore, Zhu et al. [41] reported that *miR-100* rs1834306 A>G may decrease the risk for congenital Hirschsprung disease in a Chinese pediatric population.

According to the predication from SNPinfo, *miR-100* rs1834306 is a binding sites of transcription factors. The studies described above indicate that the *miR-100* gene may participate in BA pathogenesis via complex regulatory networks. Therefore, we believe that *miR-100* might modulate cell apoptosis, proliferation, and differentiation via the notch signaling pathway [42]. On the other hand, *miR-100* might also activate mTOR to control cellular growth, metabolism, and immunity [43], and further promote the progression of BA.

Although our study revealed a correlation between the *miR-100* rs1834306 A>G polymorphism and biliary atresia risk, it does has some limitations that should be mentioned. First, only one polymorphism in the *miR-100* gene was selected for this study, and the combined effects of *miR-100* rs1834306 A>G in combination with other polymorphisms in related genes on BA progression were not studied. Second, as a hospital-based retrospective investigation, a certain degree of admission bias, such as information gathering and population selection, is unavoidable. Third, lifestyle and environmental factors were not taken in account, and those factors may hide the real association between *miR-100* and BA. Fourth, our conclusion cannot be speculated outward into other ethnicities, because all our subjects were recruited from the Han population. Fifth, more functional experiments are required to confirm the outcome of this investigation.

In summary, we found that the *miR-100* rs1834306 A>G polymorphism is in association with an increased risk for biliary atresia. However, our results need to be further verified in future investigations with more sample sizes and more diverse subject ethnic groups.

## Figures and Tables

**Table 1:** Association between the *miR-100* rs1834306 A>G polymorphism and biliary atresia susceptibility.

Genotype	Cases (*n* = 484)	Controls (*n* = 1445)	*P* ^a^	Crude OR (95% CI)	*P*	Adjusted OR (95% CI)^b^	*P* ^b^
rs1834306 A>G (HWE = 0.126)							
AA	150 (30.99)	480 (33.22)		1.00		1.00	
AG	241 (49.79)	730 (50.52)		1.06 (0.84-1.34)	0.646	1.05 (0.81-1.37)	0.699
GG	93 (19.21)	235 (16.26)		1.27 (0.94-1.71)	0.126	**1.44 (1.02-2.03)**	**0.041**
Additive			0.151	1.12 (0.96-1.30)	0.152	1.17 (0.99-1.39)	0.065
Dominant	334 (69.01)	965 (66.78)	0.366	1.11 (0.89-1.38)	0.366	1.14 (0.89-1.46)	0.307
Recessive	391 (80.79)	1210 (83.74)	0.135	1.23 (0.94-1.60)	0.135	**1.39 (1.02-1.89)**	**0.036**

Notes: the results are in bold, if the 95% CI excluded 1 or the *P* values less than 0.05. ^a^*χ*^2^ test for genotype distributions between biliary atresia patients and control. ^b^Adjusted for age and gender. Abbreviations: OR: odds ratio; CI: confidence interval; HWE:Hardy-Weinberg equilibrium.

## Data Availability

All data used and analyzed during this study are available from the corresponding author on reasonable request.
